# Cancer-specific survival and metastasis in pancreatic mucinous cystadenocarcinoma: A SEER-based cohort study

**DOI:** 10.3389/fonc.2022.985184

**Published:** 2022-10-20

**Authors:** Ruobing Wang, Dan Su, Yueze Liu, Jiangdong Qiu, Zhe Cao, Gang Yang, Wenhao Luo, Jinxin Tao, Taiping Zhang

**Affiliations:** ^1^ Department of General Surgery, Peking Union Medical College Hospital, Chinese Academy of Medical Sciences and Peking Union Medical College, Beijing, China; ^2^ Clinical Immunology Center, Chinese Academy of Medical Sciences and Peking Union Medical College, Beijing, China

**Keywords:** pancreatic mucinous cystadenocarcinoma, survival, metastasis, nomogram, SEER database

## Abstract

**Aims:**

This study aimed to investigate the prognostic value of clinical features for cancer-specific survival (CSS) and metastasis in patients with pancreatic mucinous cystadenocarcinoma (MCAC). We further constructed and validated an effective nomogram to predict CSS.

**Methods:**

We screened patients diagnosed with pancreatic MCAC from Surveillance Epidemiology and End Results (SEER) database. Kaplan-Meier curves were used to determine the CSS time. Univariate and multivariate Cox and logistic regression analyses were conducted to identify the prognostic factors for CSS and metastasis. The nomogram was constructed to predict the prognosis of pancreatic MCAC based on the results from the multivariate analysis. We used the concordance index (C-index), the area under the curve (AUC), and the calibration plots to determine the predictive accuracy and discriminability of the nomogram.

**Results:**

Multivariate Cox analysis revealed that age, primary site, grade, and radiotherapy were independent prognostic factors associated with CSS. Multivariate logistic regression analysis revealed that surgery and grade were independent risk factors associated with metastasis. The independent risk factors were included to construct a prognosis prediction model for predicting CSS in patients with pancreatic MCAC. The concordance index (C-index), receiver operating characteristic (ROC) curves, and calibration plots of the training cohort and the validation cohort showed that the nomogram had an acceptable predictive performance.

**Conclusion:**

We established a nomogram that could determine the 3- and 5-year CSS, which could evaluate individual clinical outcomes and provide individualized clinical decisions.

## Introduction

In recent years, the overall prevalence of pancreatic cystic tumors (PCN) has increased with widespread use of cross-sectional imaging modalities and advanced screening techniques (1). Not only have more accidental PCN been found in the past decade, but they are also smaller when identified (2, 3). In 2000, the World Health Organization (WHO) histological classification of PCN outlined four general categories: serous cystic neoplasm (SCN), mucinous cystic neoplasm (MCN), intraductal papillary mucinous neoplasm (IPMN), and solid pseudopapillary neoplasm (SPN) (4).

Among them, MCN and IPMN are known as precancerous lesions, and surgical resection should be considered if the malignant transformation occurs (5, 6). For the MCN, it is classified according to the degree of dysplasia into mucinous cystadenoma, mucinous cystic neoplasms with moderate dysplasia, and mucinous cystadenocarcinoma (MCAC) (7, 8). Although the pancreatic MCAC has been reported to have a better prognosis than pancreatic ductal adenocarcinoma, the median follow-up was only 27 months, with a 3-year overall survival rate of 59% and a recurrence-free survival rate of 64% (5). To avoid incomplete treatment of pancreatic MCAC, a standard oncologic resection with lymph node dissection was recommended (9).

Distant metastasis is one of the characteristics of malignant tumors. Previous studies showed that the observed survival time of pancreatic MCAC patients with distant metastasis was only 4 months (10). The analysis of the risk factors for pancreatic MCAC metastasis is essential. However, the studies on pancreatic MCAC with distant metastasis are limited, and meanwhile, there are few studies on the effects of metastasis and other factors on pancreatic MCAC survival. Therefore, we aimed to evaluate the prognostic value of clinical features for metastasis and cancer-specific survival (CSS) in this retrospective study.

We analyzed and compared the prognostic features of pancreatic MCAC based on the cases collected from the Surveillance, Epidemiology, and End Results (SEER) database. Nomogram is a reliable and convenient statistical prognostic model widely used to estimate the probability of events such as cancer prognosis and recurrence (11–13). To our knowledge, no model has been established to predict the survival and metastasis of patients with pancreatic MCAC. Therefore, we intend to establish nomograms to predict 3-year and 5-year CSS and the metastasis for pancreatic MCAC based on significant prognostic factors.

## Materials and methods

### Study design and patient selection

**Figure 1:**
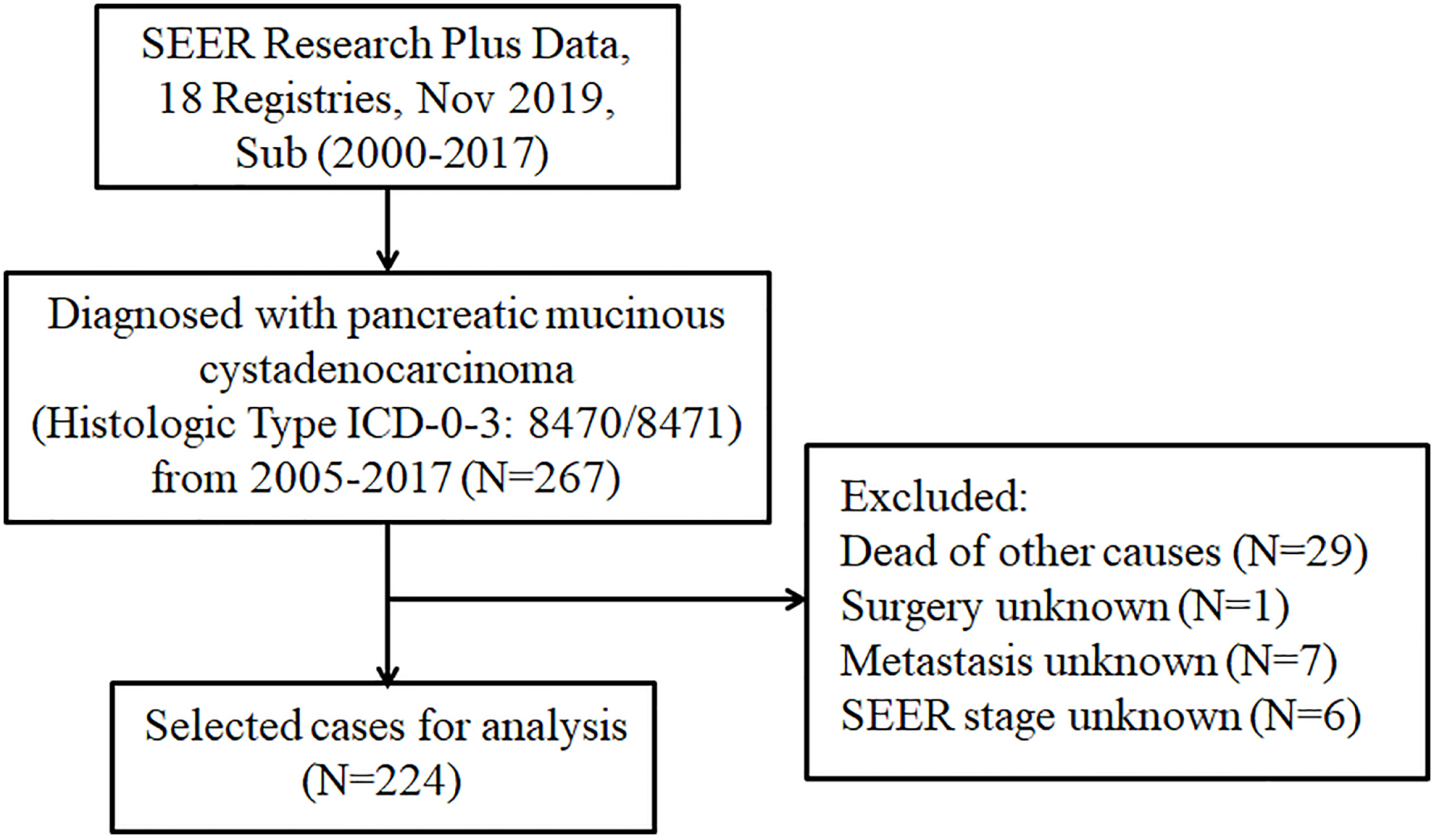
The flowchart displaying the selection procedure of cases in the SEER database. SEER, Surveillance Epidemiology and End Results.

The SEER database is the largest clinical dataset in the United States, providing free data on cancer incidence and survival (14). For this retrospective study, the data of patients with pancreatic MCAC were obtained from the SEER database. The incidence SEER Research Plus Data, 18 Registries, Nov 2019, Sub (2000–2017) was employed as the data source. The cases were extracted from SEER*Stat Database (version 8.4.0.1) through “SEER Site Recode” using the term “pancreas”, along with the Histologic Type ICD-O-3 as 8470/8471. We collected data on the following characteristics of patients with pancreatic MCAC: Age at diagnosis, gender, race, primary site, grade, SEER stage, tumor size, surgery, chemotherapy, radiotherapy, metastasis, marital status, and follow-up information. Overall survival (OS) is defined as time from randomization to death due to any cause. In this study, cancer-specific survival is defined as the time from randomization to death as a result of pancreatic MCAC. We excluded patients for whom the survival information and detailed clinical characteristics were not available. The flowchart of the selection procedure for this study was shown in **Figure 1**.

### Statistical analysis

Statistical analyses were performed using SPSS (version 24.0 IBM Corporation, USA) and R software (version 4.2.0). Kaplan-Meier analysis was performed to compare the differences in CSS between different subgroups based on the log-rank test using the survival package in R. Univariate Cox proportional hazards model was used to select the potential prognostic factors by calculating the hazard ratio (HR) and 95 percent confidence intervals (CIs). The variables with a p<0.15 were incorporated into multivariate Cox analysis to determine the independent prognostic factors related to clinical prognosis in pancreatic MCAC patients. All variables were further analyzed by univariate logistic regression analysis by calculating the odd ratio (OR) and 95 percent CIs. The variables with a p<0.15 were incorporated into multivariate logistic analysis to determine the risk factors for metastasis in pancreatic MCAC patients. P value <0.05 was considered to be statistically significant. Nomogram was constructed based on the independent prognostic factors in multivariate Cox analysis (p value<0.05) to predict the CSS (15). Concordance index (C-index) and the area under the ROC curve (AUC) were used to evaluate and compare the precision of predicting 3- and 5-year CSS with the nomogram. The calibration plot was used to compare the mean predicted 3- and 5-year CSS rate with the mean actual 3- and 5-year CSS rate through Kaplan–Meier analysis.

## Results

### Clinical characteristics

A total of 224 pancreatic MCAC cases diagnosed between 2005 and 2017 from the SEER database were included in this study. Patients were diagnosed at the age of more than 70 (40.6%), female (68.7%), white (76.8%), and married (53.6%). Regarding the primary site, 60 (26.8%) patients with tumors located in the pancreatic head. Among the 224 patients with pancreatic MCAC grade analyzed for incidence, 16.1%, 24.6%, and 9.8% were well differentiated, moderately differentiated, and poorly differentiated/undifferentiated, respectively. Regarding the tumor size, 96 cases (42.9%) with a size larger than 5cm. In terms of treatment, 145 cases (64.7%) underwent surgery, 82 cases (36.5%) underwent chemotherapy, and 38 cases (17%) underwent radiotherapy. The distant metastasis rate was 18.3% in patients with pancreatic MCAC. Demographic and clinicopathological characteristics of patients with pancreatic MCAC were summarized in **Table 1**.

**Table 1:** Clinical characteristics of patients with pancreatic mucinous cystadenocarcinoma.

Clinical characteristics	Total N=224 (%)
Age	
<70	133 (59.4)
≥70	91 (40.6)
Gender	
Male	70 (31.3)
Female	154 (68.7)
Race	
White	172 (76.8)
Black	30 (13.4)
Other	22 (9.8)
Site	
Head of pancreas	60 (26.8)
Other	164 (73.2)
Grade	
I	36 (16.1)
II	55 (24.6)
III+IV	22 (9.8)
Unknown	111 (49.5)
SEER stage	
Localized/Regional	179 (80.0)
Distant	45 (20.0)
Tumor size	
≤5cm	101 (45.1)
>5cm	96 (42.9)
Unknown	27 (12.0)
Surgery	
No	79 (35.3)
Yes	145 (64.7)
Chemotherapy	
No	142 (63.4)
Yes	82 (36.5)
Radiotherapy	
No	186 (83.0)
Yes	38 (17.0)
Metastasis	
No	183 (81.7)
Yes	41 (18.3)
Marital status	
Married	120 (53.6)
Single	36 (16.1)
Divorced or Separated	19 (8.5)
Widowed	38 (17.0)
Unknown	11 (4.9)

SEER, Surveillance Epidemiology and End Results.

### Survival analysis

**Figure 2:**
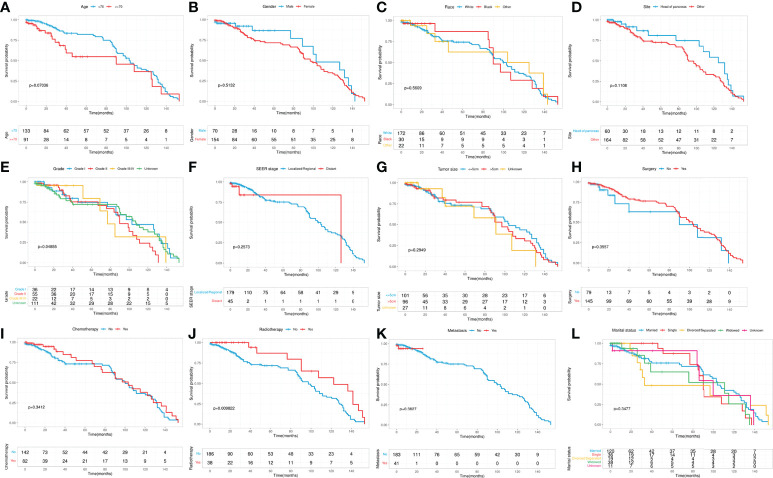
Kaplan-Meier curves stratified by patient characteristics: **(A)** Age; **(B)** Gender; **(C)** Race; **(D)** Primary site; **(E)** Grade; **(F)** SEER stage; **(G)** Tumor size; **(H)** Surgery; **(I)** Chemotherapy; **(J)** Radiotherapy; **(K)** Metastasis; **(L)** Marital status.

Kaplan-Meier curves were performed to compare the differences in CSS on age, gender, race, primary site, grade, SEER stage, tumor size, surgery, chemotherapy, radiotherapy, metastasis, and marital status. The results showed that significant differences in CSS on grade and radiotherapy (**Figure 2**). To further identify the prognostic factor associated with survival of patients with pancreatic MCAC, Cox regression analysis was conducted. Univariate Cox analysis was used to screen the potential prognostic factors from these 12 factors. The multivariate Cox analysis revealed that the age (HR=1.789, 95% CI=1.016-3.151, *P*=0.044), primary site (HR=2.079, 95% CI=1.104-3.918, *P*=0.024), grade (II: HR=1.921, 95% CI=1.007-3.663, *P*=0.048), radiotherapy (HR=0.450, 95% CI=0.240-0.843, *P*=0.013) were independent prognostic factors for CSS in patients with pancreatic MCAC (**Table 2**).

**Table 2:** Univariate and multivariate Cox analysis of cancer-specific survival in patients with pancreatic mucinous cystadenocarcinoma from SEER.

Variables	Univariate analysis		Multivariate analysis	
	HR (95% CI)	*P*	HR (95% CI)	*P*
Age				
<70	Reference		Reference	
≥70	1.543 (0.941-2.53)	0.086	1.789 (1.016-3.151)	**0.044**
Gender				
Male	Reference			
Female	1.204 (0.676-2.143)	0.529		
Race				
White	Reference			
Black	0.877 (0.463-1.662)	0.687		
Other	0.866 (0.430-1.741)	0.686		
Site				
Head of pancreas	Reference		Reference	
Other	1.507 (0.888-2.555)	0.128	2.079 (1.104-3.918)	**0.024**
Grade				
I	Reference		Reference	
II	1.745 (0.928-3.283)	0.084	1.921 (1.007-3.663)	**0.048**
III+IV	1.220 (0.507-2.935)	0.656	1.214 (0.503-2.929)	0.666
Unknown	1.077 (0.625-1.855)	0.790	1.459 (0.811-2.626)	0.208
SEER stage				
Localized/Regional	Reference			
Distant	1.817 (0.633-5.215)	0.267		
Tumor size				
≤5cm	Reference			
>5cm	1.172 (0.764-1.798)	0.467		
Unknown	1.452 (0.643-3.278)	0.369		
Surgery				
No	Reference			
Yes	0.725 (0.377-1.394)	0.335		
Chemotherapy				
No	Reference			
Yes	0.803 (0.511-1.262)	0.341		
Radiotherapy				
No	Reference		Reference	
Yes	0.470 (0.259-0.855)	0.013	0.450 (0.240-0.843)	**0.013**
Metastasis				
No	Reference			
Yes	1.987 (0.416-9.501)	0.390		
Marital status				
Married	Reference			
Single	1.204 (0.647-2.240)	0.558		
Divorced/Separated	0.902 (0.453-1.798)	0.770		
Widowed	1.297 (0.633-2.660)	0.478		
Unknown	1.081 (0.462-2.527)	0.858		

HR, hazard ratio; CI, confidence interval; SEER, Surveillance Epidemiology and End Results.

Bold values mean P value <0.05.

### Construction and validation of the nomogram for CSS

**Figure 3:**
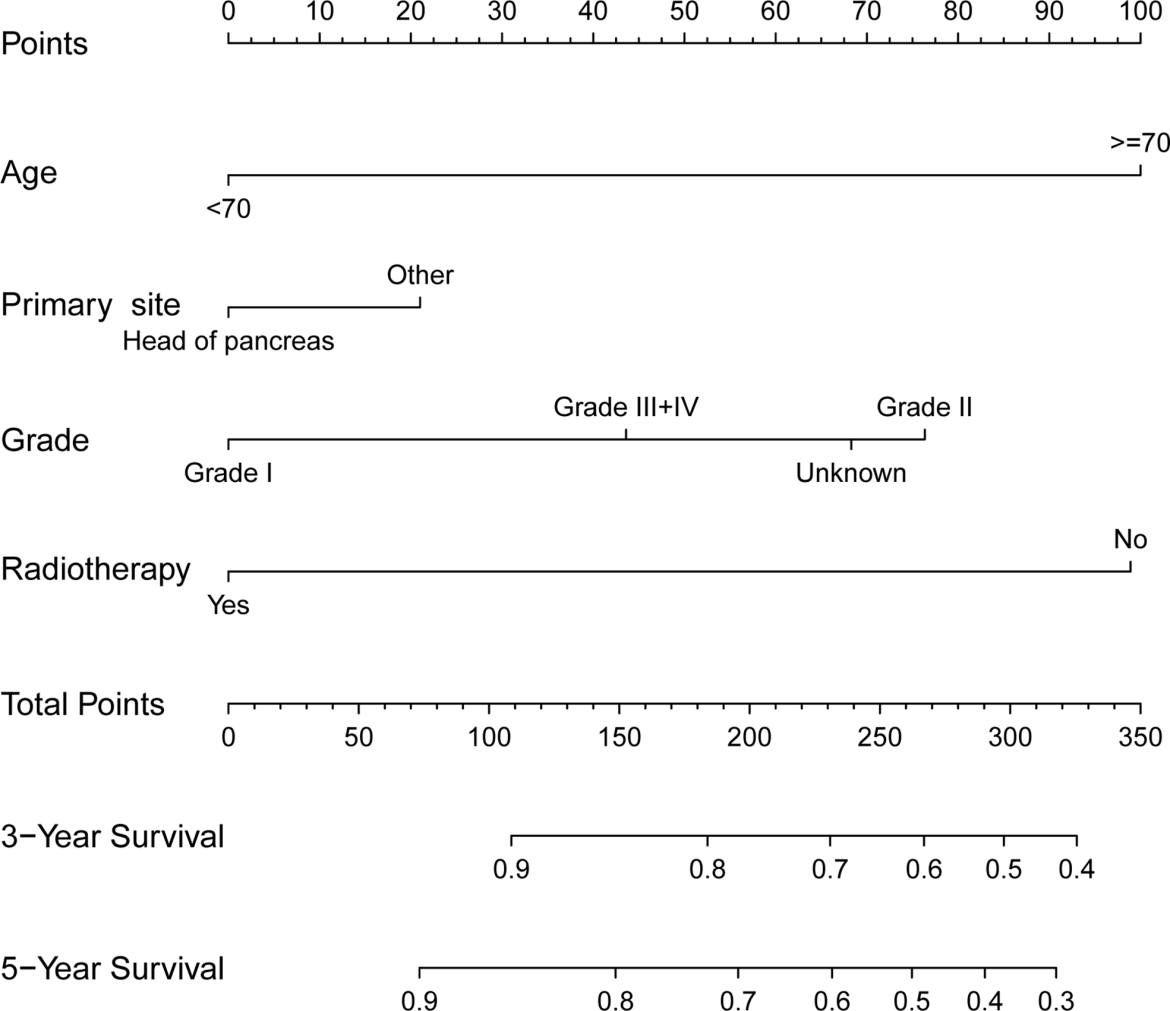
Prognostic nomogram predicting 3- and 5-year cancer-specific survival probability for patients with pancreatic mucinous cystadenocarcinoma. Summarizing the scores of each variable together and the total scores are located on the Total Points axis. Draw a vertical line down to the survival axis to determine the probability of 3- and 5-year cancer-specific survival.

**Figure 4:**
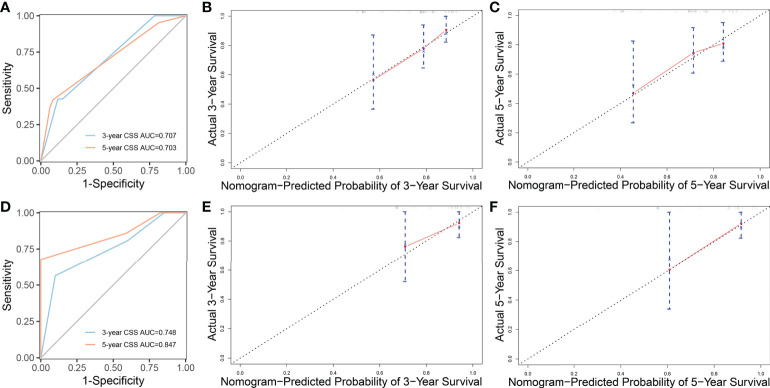
ROC curves and calibration plots of nomogram. **(A)** ROC curves of nomogram to predict 3- and 5-year cancer-specific survival in the training cohort. **(B, C)** Calibration plots of the nomogram for 3- and 5-year cancer-specific survival in the training cohort. **(D)** ROC curves of nomogram to predict 3- and 5-year cancer-specific survival in the validation cohort. **(E, F)** Calibration plots of the nomogram for 3- and 5-year cancer-specific survival in the validation cohort. ROC curves, receiver operating characteristic curves.

The whole cohort was divided into the training cohort (70%) and the validation cohort (30%). We used the training cohort and validation cohort to construct and validate the nomogram for CSS, respectively. Based on the independent prognostic factors in the multivariable Cox regression analysis, we constructed a nomogram to predict the probabilities of CSS at 3 and 5 years (**Figure 3**). The C-index of training cohort and validation cohort were 0.676 (95% CI=0.592-0.760) and 0.664 (95% CI=0.500-0.830). The ROC curves were used to compare the precision of the 3- and 5-year CSS predictions. For the training cohort, the AUC values of the nomogram that predicted 3- and 5-year CSS rates were 0.707 and 0.703 (**Figure 4A**). By observing the calibration plots, the results were highly consistent with the predicted results (**Figures 4B, C**). For the validation cohort, the AUC values of the nomogram that predicted 3- and 5-year CSS rates were 0.748 and 0.847 (**Figure 4D**). The calibration plots also demonstrated the consistency between nomogram predictions and actual observations (**Figures 4E, F**).

### Risk factors of metastasis

To identify the risk factors associated with metastasis in patients with pancreatic MCAC, univariate and multivariate logistic regression analyses were conducted. The results from univariate logistic regression analysis showed that grade, surgery, radiotherapy, and marital status were associated with metastasis. The multivariable logistic regression analysis indicated that surgery (OR=0.109, 95% CI=0.036-0.329, *P*<0.001) and grade (II: OR=0.148, 95% CI=0.026-0.832, *P*=0.030) were the independent risk factors related to metastasis. These results were shown in **Table 3**. Since there were only two independent risk factors for metastasis, we could not construct a nomogram for prediction.

**Table 3:** Univariate and multivariate logistic regression analysis of metastasis in patients with pancreatic mucinous cystadenocarcinoma from SEER.

Variables	Univariate analysis		Multivariate analysis	
	OR (95% CI)	*P*	OR (95% CI)	*P*
Age				
<70	Reference			
≥70	1.502 (0.761-2.968)	0.241		
Gender				
Male	Reference			
Female	0.745 (0.366-1.514)	0.416		
Race				
White	Reference			
Black	1.137 (0.429-3.016)	0. 796		
Other	1.011 (0.320-3.196)	0. 985		
Site				
Head of pancreas	Reference		Reference	
Other	1.980 (0.826-4.745)	0.125	2.000 (0.746-5.360)	0.168
Grade				
I	Reference		Reference	
II	0.156 (0.030-0.802)	0.026	0.148 (0.026-0.832)	**0.030**
III+IV	0.414 (0.078-2.204)	0.301	0.431 (0.074-2.521)	0.350
Unknown	1.534 (0.608-3.872)	0.365	0.382 (0.107-1.370)	0.140
Tumor size				
≤5cm	Reference			
>5cm	1.533 (0.720-3.264)	0.267		
Unknown	2.617 (0.962-7.116)	0.060		
Surgery				
No	Reference		Reference	
Yes	0.134 (0.062-0.288)	<0.001	0.109 (0.036-0.329)	**<0.001**
Chemotherapy				
No	Reference			
Yes	1.455 (0.732-2.894)	0.285		
Radiotherapy				
No	Reference		Reference	
Yes	0.209 (0.048-0.908)	0.037	0.316 (0.066-1.508)	0.148
Marital status				
Married	Reference		Reference	
Single	2.500 (1.017-6.145)	0.046	2.044 (0.731-5.714)	0.173
Divorced/Separated	0.361 (0.045-2.894)	0.337	0.246 (0.026-2.347)	0.337
Widowed	2.321 (0.950-5.673)	0.065	1.358 (0.488-3.781)	0.557
Unknown	3.714 (0.976-14.135)	0.054	4.971(0.956-25.854)	0.057

OR, odd ratio; CI, confidence interval; SEER, Surveillance Epidemiology and End Results.

Bold values mean P value <0.05.

## Discussion

MCN is one of the pancreatic cystic tumors, characterized by a large, isolated, separated, thick-walled cyst containing mucin or a mixture of mucin and hemorrhagic material (16). Due to the potential for malignant transformation of MCN, some consensus guidelines recommend surgical resection. In 2018, the European Study Group suggests that patients with MCN who are asymptomatic, without mural nodules, and less than 40mm in size can be followed by magnetic resonance imaging (MRI), endoscopic ultrasonography (EUS), or a combination of both (9). However, for MCN with features indicating high-grade dysplasia or cancer, surgical resection is recommended. Pancreatic mucinous cystadenocarcinoma, as a malignant MCN, has a reported prevalence of 17.5% (17).

Given the rarity of this neoplasm, few studies have reviewed the cases of pancreatic MCAC. As the largest clinical database in the United States, the SEER database compensates for the low number of pancreatic MCAC cases and provides detailed clinical data including demographic characteristics, survival time, and the distant metastasis. By analyzing the data collected from the SEER database, we intend to establish nomogram models to predict the survival and metastasis in pancreatic MCAC patients.

We first compared the differences in CSS on clinical characteristics. The results showed significant differences in CSS on grade and radiotherapy. In the Cox regression analysis, we identified that the age, primary site, grade, and radiotherapy were independent prognostic factors for CSS in patients with pancreatic MCAC. Age and grade have been found to be independent factors for cancer prognosis in many studies (18–20). At the primary site, the tumors at the head of pancreas have a better prognosis than those at other sites. For nonsurgical treatment, standard chemotherapy and radiotherapy for pancreatic MCAC have not been established. This kind of malignant tumor was sometimes treated as pancreatic duct adenocarcinoma (21–23). Our results indicated that pancreatic MCAC patients who underwent radiotherapy have a better prognosis.

However, a previous study showed that surgery and tumor stage were independent prognostic factors of CSS (10). In this study, our results did not show that surgery and tumor stage were independent factors, which may be related to the fact that the previous study analyzed 2-year CSS only. Due to the cut-off of age was 50 and there was no analysis for radiotherapy, these two were not found as independent prognostic factors. Moreover, records in that study were from patients diagnosed from 1988 to 2012 in the SEER database. All these reasons probably contribute to the discrepant results between studies.

Although the multivariable Cox analysis in our study identified age, primary site, grade, and radiotherapy were independent prognostic factors for CSS, these variables could not predict survival alone. Specific and clinically applicable nomograms can accurately assess the prognosis of patients. However, there is no prognostic model for pancreatic MCAC. Our study filled the gap by establishing a nomogram to predict the survival of pancreatic MCAC based on a large dataset. The nomogram we established in this study had a good predictive value. The ROC curves indicated that the nomogram possessed considerable predictive power. Moreover, the calibration plots of the nomogram revealed a strong consistency between actual observation and prediction. In clinical practice, the nomogram can objectively evaluate individual clinical outcomes and guide doctors in determining the most appropriate treatment strategy for patients.

It is widely believed that metastasis is closely associated with poor outcomes of malignant tumors. The 5-year relative survival rate of pancreatic cancer is 11.5%, but for the pancreatic cancer with distant metastasis is 3.1% (24). Although the metastasis was not an independent prognostic factor for CSS in this study, it still had an adverse effect on prognosis of patients. We further investigated the risk factors associated with metastasis in patients with pancreatic MCAC. Our results showed that grade, surgery, radiotherapy, and marital status were associated with metastasis. The multivariable logistic regression analysis demonstrated that surgery and grade were independent risk factors for metastasis.

Surgery has been reported as the risk factor for metastasis in many cancers (12, 25). We found that the risk of metastasis decreased significantly in patients who underwent surgery. Even though the improvement in CSS was not statistically significant, surgery had a positive effect on patients with pancreatic MCAC (26). Interestingly, there was a lower probability of metastasis in grade II compared with grade I. As a matter of common sense, grade II should be more malignant than grade I, the results seemed to be strange. However, we speculated that grade II patients had a lower risk of metastasis than grade I patients due to their poor prognosis and death prior to metastasis. The results might also be related to the high proportion of “Unknown” status in grade, which should be investigated in further study. For marital status, it has been reported as the risk factor for the survival of metastatic pancreatic ductal adenocarcinoma and metastatic bladder cancer (27, 28). In our research, marital status was associated with metastasis, but it was not the independent risk factor for CSS and metastasis for MCAC. Unfortunately, there were not enough independent risk factors for metastasis, so we could not construct a nomogram for predicting metastasis.

The main limitation of this study is the rarity of pancreatic MCC cases. Although this has been a large sample size study for pancreatic MCAC survival and metastasis, the overall sample size is still small. In addition, we did not conduct external validation in our central database due to insufficient samples of pancreatic MCAC. The current nomogram needs more cohorts for validation. There are other potential limitations in our study. First, selection bias may occur in retrospective studies when the selection criteria are associated with the risk factors being investigated (29). Besides, this study relied on the SEER database, which meant that some potential predictors not included in the database could not be involved in the nomogram.

This is the first multicenter retrospective study to establish a nomogram predicting CSS for pancreatic MCAC patients and to analyze independent factors of metastasis. Cox regression analysis revealed that age, primary site, grade, and radiotherapy were independent prognostic factors for CSS. Further, surgery and grade were identified as independent risk factors for metastasis. A nomogram for the estimation of 3- and 5-year CSS was established based on the SEER database. ROC curves and calibration plots indicated the considerable predictive power for the nomogram. Our results can be used to evaluate individual clinical outcomes and provide individualized clinical decisions for future treatment strategies and patient management.

## Data availability statement

The original contributions presented in the study are included in the article. Further inquiries can be directed to the corresponding author.

## Author contributions

RW participated in the design of this study and wrote the manuscript. TZ conceived the original idea and supervised the project. DS, YL, JQ, and ZC collected the data and performed the statistical analysis. GY, WL, and JT reviewed the literature and revised the manuscript. All authors contributed to the article and approved the submitted version.

## Funding

This study was supported by grants from the National Natural Science Foundation of China (No.81972258, No. 82103016), National Key R&D Program of China (2018YFE0118600), China Postdoctoral Science Foundation (2021T140071 and 2021M690462), National Multidisciplinary Cooperative Diagnosis and Treatment Capacity Building Project for Major Diseases.

## Acknowledgments

We thank the National Cancer Institute for providing the Surveillance, Epidemiology, and End Results (SEER) database.

## Conflict of interest

The authors declare that the research was conducted in the absence of any commercial or financial relationships that could be construed as a potential conflict of interest.

## Publisher’s note

All claims expressed in this article are solely those of the authors and do not necessarily represent those of their affiliated organizations, or those of the publisher, the editors and the reviewers. Any product that may be evaluated in this article, or claim that may be made by its manufacturer, is not guaranteed or endorsed by the publisher.
